# Effect of a Traditional Herbal Prescription, Kyung-Ok-Ko, on Male Mouse Spermatogenic Ability after Heat-Induced Damage

**DOI:** 10.1155/2015/950829

**Published:** 2015-10-11

**Authors:** Deok-Sang Hwang, Hyo Geun Kim, Sodam Park, Nam Doo Hong, Jong Hoon Ryu, Myung Sook Oh

**Affiliations:** ^1^Department of Korean Medicine Obstetrics & Gynecology, College of Korean Medicine, Kyung Hee University, 26 Kyungheedae-ro, Dongdaemun-gu, Seoul 130-701, Republic of Korea; ^2^Department of Life and Nanopharmaceutical Science, Graduates School and Kyung Hee East-West Pharmaceutical Research Institute, Kyung Hee University, 26 Kyungheedae-ro, Dongdaemun-gu, Seoul 130-701, Republic of Korea; ^3^Department of Oriental Pharmaceutical Science, College of Pharmacy, Kyung Hee University, 26 Kyungheedae-ro, Dongdaemun-gu, Seoul 130-701, Republic of Korea; ^4^R&D Center, Kwang Dong Pharmaceutical Co., Ltd., 621-1 Jangdang-dong, Pyongtaek-si, Kyonggi-do 459-020, Republic of Korea

## Abstract

Kyung-Ok-Ko (KOK), a well-known traditional Korean medicinal formula, has long been used to invigorate the essential *qi*. This use of KOK may be associated with reproductive ability as a more modern concept. The protective effect of KOK was evaluated against deterioration of testicular function induced by heat exposure in male mice. Male fertility was disrupted by scrotal heat stress at 43°C for 5 weeks. KOK (0.25, 0.50, and 2.00 g/kg/day) was administered orally at 3 h after the stress. To evaluate the protective effect of KOK, body weight, testicular weight, sperm count, sperm motility, and histopathological changes in the testes were evaluated. KOK-treated mice significantly recovered their general health, as evidenced by body weight. KOK-treated mice also showed significantly higher testes weights, sperm counts, and sperm motility than did the heat stress group. KOK-treated mice significantly recovered the morphological appearance of the seminiferous tubules and seminiferous epithelium. Furthermore, KOK-treated mice significantly increased antioxidant enzyme activities and reduced the protein expressions of apoptosis in the testes. KOK significantly protects against heat-induced damage to testicular function in male mice by inhibiting oxidative stress and apoptosis, indicating that KOK may be an effective agent for treatment of heat-induced male infertility.

## 1. Introduction 

Infertility affects 15% of couples worldwide. The male contribution is 45–50%, and males are the sole cause in 20% of cases [1, 2]. The causes of male infertility are multifactorial which include anatomical and genetic defects, testicular injury and disease, sperm disorders, hormonal dysfunction, aging, and environmental- and lifestyle-related factors [3–5]. Among environmental and lifestyle factors, genital heat stress appears to be a major contributor to impairment of male reproductive health [6]. For example, sitting in a hot bath or car for a long time is associated with significantly higher scrotal temperatures, resulting in downregulation of spermatogenesis with declines in semen volume, sperm motility, and sperm morphology [3–5].

Kyung-Ok-Ko (KOK), also known as Qiong-yu-gao in China, is a traditional Korean medicinal formula composed of* Rehmannia glutinosa *var.* purpurea*,* Panax ginseng*,* Poria cocos*,* Lycium chinense*,* Aquilaria agallocha*, and honey [7, 8]. KOK has long been used to maintain health and increase longevity [8]. Based on traditional medicine physiology, male infertility is closely related to kidney storage of the essence* qi*, which corresponds roughly to the modern concept of the male gametes [9]. Therefore, herbal prescriptions that increase the essential* qi* have been used to treat impotence and sterility secondary to male infertility. KOK contains phytochemicals, such as valine, aspartic acid, and arginine, which are antioxidants and exhibit oxidase inhibition, tyrosinase inhibition, nitric oxide inhibition, and superoxide dismutase-like activities [9]. In addition, some studies have examined the effects of KOK on age-related disorders as well as the biological properties of KOK, including its antioxidant, anti-inflammatory, antifatigue, and immunological activities [9, 10]. These biological properties are associated with medical therapy that aims to improve sperm parameters in male infertility [11–13].

Based on the above mentioned effects of KOK and its use in traditional medicine, and previous reports, it is hypothesized that KOK might be effective to treat male infertility. Hence, the aim of this study is to evaluate the effects of KOK on sperm quality parameters, such as sperm count, sperm motility, and testicular weight, as well as the histopathology, antioxidant, and apoptotic changes in male mice with infertility induced by heat exposure.

## 2. Materials and Methods

### 2.1. Materials

M199 medium was purchased from Gibco Industries, Inc. (Auckland, NZ). Phosphate-buffered saline (PBS), sodium chloride (NaCl), bovine serum albumin (BSA), hematoxylin, and eosin were purchased from Sigma-Aldrich (St. Louis, MO, USA). Tetramethylethylenediamine, protein assay kit, tween 20, ammonium persulfate, acrylamide, ECL reagent, and skim milk were purchased from Bio-Rad Lab. (Hercules, CA, USA). B-cell lymphoma-associated X protein (Bax), cytochrome c, and *β*-actin antibodies were obtained from Santa Cruz Biotechnology, Inc., (Delaware Avenue, CA, USA). Cleaved caspase-3 and HRP-conjugated secondary antibodies were purchased from Cell Signaling Technology (Beverly, MA, USA). The total glutathione (GSH) assay kit and the oxidized glutathione (GSSG)/GSH Quantification Kit were purchased from Dojindo Molecular Tech. (Tokyo, Japan). KOK was the same as that used in the previous study [14] in which chemical profiling and standardization of KOK had been performed and KOK (Lot No. SU12) was donated by Kwang Dong Pharmaceutical Co. (Pyongtaek, Korea).

### 2.2. Animals and Heat Exposure

Male ICR mice (7 weeks, 30–32 g) were purchased from Daehan Biolink (Eumseong, Korea). The mice were divided randomly into five groups of eight mice each: (1) normal group, (2) heat exposure (HE) group, (3) HE + KOK 0.25 g group, (4) HE + KOK 0.50 g group, and (5) HE + KOK 2.00 g group. The lower body, including the scrotum, in groups (2) to (5) was exposed to heat at 43°C for 10 min twice per day at 10 min intervals (6 days per week) for 5 weeks in a thermostatically controlled water bath. KOK was dissolved in distilled water and administered orally at 0.25, 0.50, or 2.00 g/kg/day, 3 h after the heat stress. The gavage doses of KOK were derived from the previous study [14, 15] and the normal group and HE group were treated with the same volume of distilled water. Four animals were housed in a single cage and had free access to water and food. The animals were kept at a constant temperature (23 ± 1°C) and humidity (60 ± 10%) and maintained under a 12 h light/dark cycle. Animal treatment and maintenance were carried out in accordance with the Principles of Laboratory Animal Care (NIH publication no. 85-23, revised 1985) and the Animal Care and Use Guidelines of Kyung Hee University, Seoul, Korea. The animals were weighed twice per week to determine the gavage volume and monitor their general health.

### 2.3. Sperm Analysis and Testes Weight

The epididymal sperm motility and count were evaluated as described in previous research with some modifications [16]. The sperm analysis was performed using a hemocytometer (Superior, Marienfeld, Germany). The mice were anesthetized with Rumpun and Zoletil solution (3 : 1 ratio, 1 mL/kg) intramuscularly on the day after the last KOK treatment. The epididymis was rapidly washed in PBS, minced in M199 medium containing 0.5% BSA, and incubated for 5 min at 37°C. Sperm were scored as motile if any movement was detected, and the total number of sperm was counted. Additionally, the entire testis from each mouse was rapidly washed in PBS and weighed. The testes were then stored at −80°C until use.

### 2.4. Histology

Frozen tissues were cut along the coronal plane (5 *μ*m) using a cryostat (Leica, Nussloch, Germany). The sections were mounted on gelatin-coated slides and stained with hematoxylin and eosin (H&E). The images were obtained using a research microscope (BX51T-32F01; Olympus Corporation, Tokyo, Japan). The effect of KOK on testicular tissue was quantified by measuring the optical density of ROIs in seminiferous tubule using the ImageJ software and the mean optical densities of each group are presented as percentages of the normal group values.

### 2.5. Total Glutathione Quantification and Oxidized Glutathione Quantification

Total GSH and GSSG levels were detected using the GSSG/GSH quantification kit with the reagent for GSH masking according to the instruction manuals. Briefly, frozen tissues were lysed in 10 mmol/L hydrochloric acid solution by freezing and thawing. To measure total GSH level, they were further treated with 5% 5-sulfosalicylic acid. 20 *μ*L coenzyme working solution, 120 *μ*L buffer solution, and 20 *μ*L enzyme working solution were added to each well at 37°C for 5 min. Then, 20 *μ*L GSH standard solution, 20 *μ*L sample solution, and 20 *μ*L substrate working solution were added for 10 min each. Absorbance was measured using a spectrophotometer at a wavelength of 405 nm, and concentrations of GSH were determined in the sample solution using a GSH standard calibration curve. To measure GSSG level, they were treated with 5% 5-sulfosalicylic acid. 40 *μ*L GSSG standard solution and 40 *μ*L sample solution with 2% masking solution each were incubated with 120 *μ*L buffer solution at 37°C for 60 min. Then, 20 *μ*L substrate working solution, 20 *μ*L coenzyme working solution, and 20 *μ*L enzyme working solution were added for 10 min each. Absorbance was measured using a spectrophotometer at a wavelength of 415 nm, and concentrations of GSSG were determined in the sample solution using a GSSG standard calibration curve.

### 2.6. Western Blotting

Frozen tissues were lysed using a protein assay kit according to the manufacturer's instructions. The lysates (protein 25 *μ*g) were separated by 10% or 12% SDS-polyacrylamide gel electrophoresis, and then transferred to a membrane. The membranes were incubated with 5% skim milk in TBST for 1 h and then with primary antibody (1 : 500 dilution) overnight at 4°C, prior to incubation with HRP-conjugated secondary antibody for 1 h. Immunoreactive bands were detected using an ECL detection kit and an LAS-4000 mini system (Fujifilm, Tokyo, Japan) was used for visualization. The intensities of the bands were normalized to the *β*-actin intensity using Multi Gauge software (Fujifilm, Tokyo, Japan).

### 2.7. Statistical Analysis

All statistical parameters were calculated using the GraphPad Prism 5.0 software (San Diego, CA). Values are expressed as the means ± standard error of the mean (SEM). Results were analyzed by one-way analysis of variance followed by Tukey's* post hoc* test. Differences with a *P* value of <0.05 were considered as statistical significance.

## 3. Results

### 3.1. Effects of KOK on Heat Exposure-Induced Reduction of Body and Testicular Weights

Mice with heat stress showed significant reduced weight compared with the control. However, KOK-treated mice subjected to heat stress recovered this reduction more efficiently than mice in the HE group (Table 1). In addition, heat stress induced a greater loss of testicular weight (weight, 47.27 ± 1.51 mg) compared to the control group (weight, 117.75 ± 2.83 mg). However, mice treated with KOK at 0.50 and 2.00 g/kg for 5 weeks showed recovery of testicular weight (79.76 ± 2.74 and 73.88 ± 4.23 mg, resp.) (Table 1).

### 3.2. Effects of KOK on Sperm Parameters against Heat Stress

To investigate the effect of KOK on the epididymal sperm count and motility, sperm parameters were measured. The sperm count of mice exposed to heat treatment was decreased significantly to 14.41% ± 0.79% of that of the control mice. However, mice treated with KOK at 0.25 to 2.00 g/kg showed an increase in sperm count to 19.85% ± 2.295 to 40.56% ± 3.22% of that of the controls. In addition, KOK-treated mice significantly recovered their sperm motility after the heat exposure to 62.73% ± 0.94% to 79.87% ± 1.32% of that of the controls, whereas heat stress reduced sperm motility in mice to 49.33% ± 2.05% of that of the controls (Table 1).

### 3.3. Effects of KOK on Histopathological Change in Testes against Heat Stress

**Figure 1:**
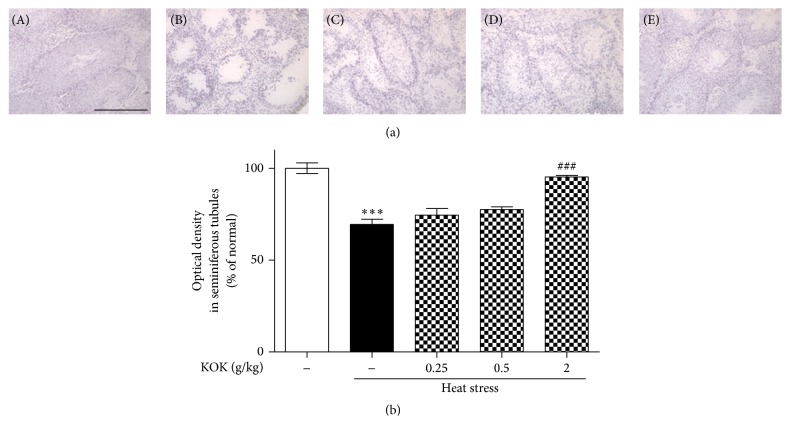
Effect of KOK on histological changes in testes of mice with heat stress-induced infertility. H&E staining was performed using testicular tissue after heat stress and/or KOK treatment for 5 weeks. Representative photomicrographs are shown in (a), and the mean optical density of seminiferous tubules was measured in (b). (A) Normal group; (B) heat stress group; (C–E) heat stress and KOK treatment at 0.25, 0.50, and 2.00 g/kg, respectively. Scale bar = 200 *μ*m. Each column represents the mean ± SEM (*n* = 6). ^*∗∗∗*^ *P* < 0.001 compared with the normal group; ^###^ *P* < 0.001 compared with the heat stress group.

To determine the effect of KOK on seminiferous tubules in testes, we performed H&E staining. A normal morphological appearance of the seminiferous tubules and spermatocytes was evident in the testes of control mice, whereas the heat-exposed testes exhibited degenerated and disorganized features and reduced spermatocyte numbers. However, KOK-treated mice significantly recovered the morphological appearance of the seminiferous tubules and seminiferous epithelium (Figure 1).

### 3.4. Effects of KOK on GSH Depletion and Apoptotic Protein Expressions in Testes against Heat Stress

**Figure 2:**
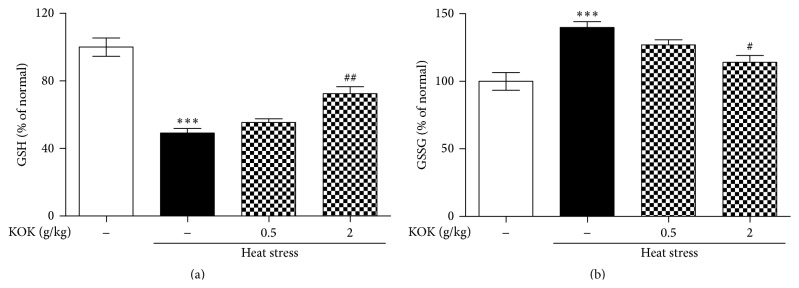
Effect of KOK on glutathione level variation in testes of mice with heat stress-induced infertility. Glutathione kit assays were performed using testicular tissue after heat stress and/or KOK treatment for 5 weeks. The total GSH level (a) and GSSG level (b) were determined. Each column represents the mean ± SEM (*n* = 4). ^*∗∗∗*^ *P* < 0.001 compared with the normal group; ^##^ *P* < 0.01,   ^#^ *P* < 0.05 compared with the heat stress group.

**Figure 3:**
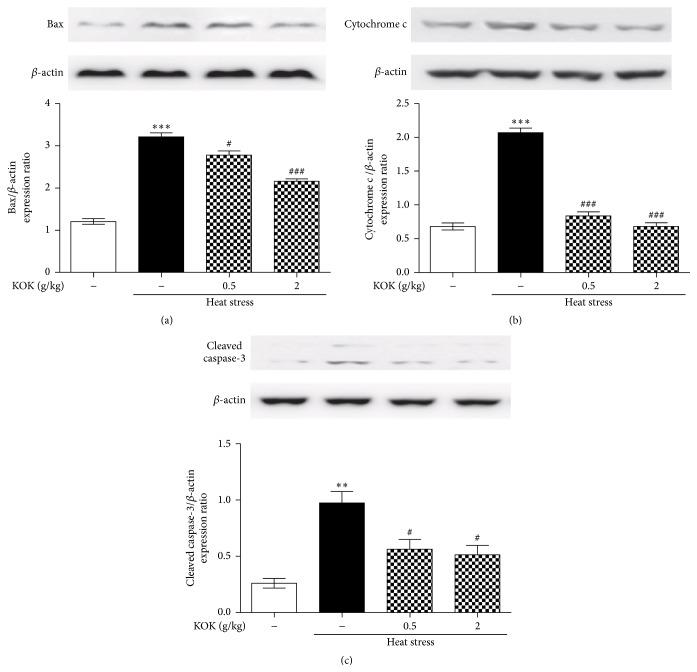
Effect of KOK on apoptotic protein expressions in testes of mice with heat stress-induced infertility. Western blotting was performed using testicular tissue after heat stress and/or KOK treatment for 5 weeks. Apoptosis factors such as Bax (a), cytochrome c (b), and cleaved caspase-3 (c) were presented. Each column represents the mean ± SEM (*n* = 3). ^*∗∗*^ *P* < 0.01, ^*∗∗∗*^ *P* < 0.001 compared with the normal group; ^#^ *P* < 0.05, ^###^ *P* < 0.001 compared with the heat stress group.

To examine the effect of KOK on heat stress-induced oxidative stress and apoptosis, the levels of total GSH and GSSH and apoptotic protein expressions were measured. In the GSH and GSSG quantification assays, treatment with heat stress reduced GSH level and increased GSSG level in the testes. However, KOK treatment recovered them (Figure 2). In addition, the testes of mice exposed to heat treatment showed increase of Bax and cytochrome c protein expressions to 265.78% ± 7.75% and 304.32% ± 9.76%, respectively, of that of the control mice. However, mice treated with KOK at 0.5 and 2.00 g/kg recovered these increases. In addition, KOK-treated mice significantly inhibited the heat treatment induced-increase in cleaved caspase-3 expression levels in the testes (Figure 3).

## 4. Discussion 

It is widely accepted that heat stress adversely affects spermatogenesis, resulting in infertility. In humans, scrotal heat treatment by occupational exposure, lifestyle, or clothing is correlated with reduced sperm concentrations, sperm motility, and normal morphology [2]. In this study, the body weight, testicular weight, sperm number, and sperm motility were reduced in male mice after heat stress, 43°C water bath for 10 min twice per day (6 days per week for 5 weeks), which corresponds with previous studies on the effect of heat stress [17–19]. However, KOK-treated mice recovered the reduction of body weights induced by heat stress more efficiently than did mice in the HE group. And, mice treated with KOK at 0.50 and 2.00 g/kg showed recovery of testicular weight from the heat-induced damage. In addition, mice treated with KOK significantly showed an increase in sperm count and sperm motility after heat exposure.

The epididymal sperm and testicular germ cells are sensitive to damage by heat stress [20]. Thus, seminiferous tubules from testes after heat stress showed pathological morphologies including degenerating cells of primarily spermatocyte origin and condensed chromatin in germ cell nuclei, resulting in disruption of spermatogenesis [21]. The present study showed that KOK-treated mice significantly recovered the morphological appearance of the seminiferous tubules and epithelium.

Generally, germ cell death and decreased sperm motility secondary to heat stress appear to be caused by oxidative stress and apoptosis which involve reactive oxygen species (ROS), the tumor suppressor protein p53, nitric oxide synthase (NOS), translocation of the proapoptotic factor Bax, release of cytochrome *c* from mitochondria, and several caspases [22, 23]. In addition, antioxidants have a significant effect on sperm oxidative stress and DNA damage in infertile patients and improve sperm motility [22, 24]. Thus, inhibition of oxidative stress and apoptosis could be protective in male infertility. In the present study, KOK treatment protected depletion of glutathione and increase of proapoptotic protein expressions in testes under heat stress condition. Taken together, these findings indicate that KOK-treated mice significantly improved male infertility induced by heat via antioxidant and antiapoptotic activities.

KOK contains ingredients that exert beneficial effects on male infertility. In a previous study, KOK treatment exerted its protective effect on polycystic ovarian syndrome induced by dehydroepiandrosterone via inhibition of inflammatory responses [15] which is also related to male fertility because infertile patients with infection have a heightened inflammatory response and parallel alterations in sperm parameters [13]. KOK was also found to inhibit the expression of IL-1*β*, a proinflammatory cytokine, thus showing anti-inflammatory properties [14]. In addition, processed rhizome of* Rehmannia glutinosa* inhibits TNF-*α* secretion by inhibiting IL-1 secretion and has anti-inflammatory activity [25].

Moreover, Ginseng Radix, the root of* Panax ginseng,* improves the motility and total number of sperm by activating cAMP-responsive element modulator [26]. Treatment with Ginseng Radix also resulted in significantly enhanced sperm counts and glial cell-derived neurotrophic factor (GDNF) mRNA and protein levels, suggesting that it induces spermatogenesis and GDNF activation in rat testes [27]. In addition, the fruit of* Lycium chinense* has been used as a traditional remedy for male infertility [28]; it possesses antioxidant activity due to its inhibition of malondialdehyde formation and activation of superoxide anion scavenging and antisuperoxide formation [29]. Furthermore, KOK possesses potential bioactive components which might protect or treat spermatogenic ability, such as valine, aspartic acid, and arginine, which are antioxidants and exhibit oxidase inhibition, nitric oxide inhibition, and superoxide dismutase-like activities [9]. Therefore, the properties of KOK and its constituent compounds, including their antioxidant, anti-inflammatory, antiapoptotic, and spermatogenesis activities, likely contributed to the effects seen in this study. We believe that some medicinal herbs may improve male fertility with relatively few side effects.

## 5. Conclusions

In this study, KOK significantly protects against heat-induced damage in male mouse testes. These results suggested that KOK may be useful for the treatment of environmental and lifestyle-related male infertility.

## Figures and Tables

**Table 1:** Body and testicular weights and sperm parameters in mice after heat stress and/or KOK treatment for 5 weeks.

Groups	Normal	HE	HE + KOK 0.25 g	HE + KOK 0.5 g	HE + KOK 2 g
Body weight (g)	35.22 ± 1.09	32.91 ± 0.49^*∗*^	31.55 ± 0.39	34.03 ± 0.76	33.18 ± 0.34
Testes weight (mg)	117.75 ± 2.83	47.27 ± 1.51^*∗∗∗*^	40.76 ± 1.04^#^	79.76 ± 2.74^###^	73.88 ± 4.23^###^
Relative testes weight (%)	0.34 ± 0.02	0.14 ± 0.01^*∗∗∗*^	0.12 ± 0.01	0.24 ± 0.01^###^	0.22 ± 0.01^###^
Sperm count (×10^6^)	61.22 ± 1.77	12.15 ± 0.49^*∗∗∗*^	40.76 ± 1.40	20.98 ± 1.35^###^	24.83 ± 1.97^###^
Sperm motility (%)	49.11 ± 0.93	24.22 ± 1.01^*∗∗∗*^	30.80 ± 0.46	36.16 ± 0.68^###^	39.22 ± 0.65^###^

The data represents the mean ± SEM (*n* = 6–8). ^*∗∗∗*^ *P* < 0.001, ^*∗*^ *P* < 0.05 compared with the normal group; ^###^ *P* < 0.001, ^#^ *P* < 0.05 compared with the heat stress group.
